# Gammaherpesvirus Co-infection with Malaria Suppresses Anti-parasitic Humoral Immunity

**DOI:** 10.1371/journal.ppat.1004858

**Published:** 2015-05-21

**Authors:** Caline G. Matar, Neil R. Anthony, Brigid M. O’Flaherty, Nathan T. Jacobs, Lalita Priyamvada, Christian R. Engwerda, Samuel H. Speck, Tracey J. Lamb

**Affiliations:** 1 Department of Microbiology and Immunology, Emory University School of Medicine, Atlanta, Georgia, United States of America; 2 Microbiology and Molecular Genetics Graduate Program, Laney Graduate School, Emory University, Atlanta, Georgia, United States of America; 3 Division of Pediatric Infectious Disease, Department of Pediatrics, Emory University School of Medicine, Emory Children’s Centre, Atlanta, Georgia, United States of America; 4 Population Biology, Ecology and Evolution Graduate Program, Laney Graduate School, Emory University, Atlanta, Georgia, United States of America; 5 Immunology and Infection Laboratory, QIMR Berghofer Medical Research Institute, Herston, Brisbane, Queensland, Australia; 6 Emory Vaccine Center, Emory University, Atlanta, Georgia, United States of America; University of Florida, UNITED STATES

## Abstract

Immunity to non-cerebral severe malaria is estimated to occur within 1-2 infections in areas of endemic transmission for *Plasmodium falciparum*. Yet, nearly 20% of infected children die annually as a result of severe malaria. Multiple risk factors are postulated to exacerbate malarial disease, one being co-infections with other pathogens. Children living in Sub-Saharan Africa are seropositive for Epstein Barr Virus (EBV) by the age of 6 months. This timing overlaps with the waning of protective maternal antibodies and susceptibility to primary *Plasmodium* infection. However, the impact of acute EBV infection on the generation of anti-malarial immunity is unknown. Using well established mouse models of infection, we show here that acute, but not latent murine gammaherpesvirus 68 (MHV68) infection suppresses the anti-malarial humoral response to a secondary malaria infection. Importantly, this resulted in the transformation of a non-lethal *P*. *yoelii* XNL infection into a lethal one; an outcome that is correlated with a defect in the maintenance of germinal center B cells and T follicular helper (Tfh) cells in the spleen. Furthermore, we have identified the MHV68 M2 protein as an important virus encoded protein that can: (i) suppress anti-MHV68 humoral responses during acute MHV68 infection; and (ii) plays a critical role in the observed suppression of anti-malarial humoral responses in the setting of co-infection. Notably, co-infection with an M2-null mutant MHV68 eliminates lethality of *P*. *yoelii* XNL. Collectively, our data demonstrates that an acute gammaherpesvirus infection can negatively impact the development of an anti-malarial immune response. This suggests that acute infection with EBV should be investigated as a risk factor for non-cerebral severe malaria in young children living in areas endemic for *Plasmodium* transmission.

## Author Summary

Nearly 1 million deaths occur annually as a result of complications associated with *P*. *falciparum* infection, with children younger than 5 being the most susceptible age group. Earlier studies have demonstrated that children co-infected with *P*. *falciparum* and Epstein-Barr virus (EBV) have impaired immune responses to control EBV, and this can result in the development of a jaw tumor called endemic Burkitt’s lymphoma (eBL). It is not known if there is any impact of acute EBV infection on the generation of anti-malarial immunity. We have used mouse models of EBV [murine gammaherpesvirus 68 (MHV68)] and malaria (*P*. *yoelii* XNL) to demonstrate that acute gammaherpesvirus infection can impair the generation of antibodies that control *Plasmodium* parasitemia, in turn causing a non-lethal *P*. *yoelii* XNL infection to become lethal. We identify a critical role for the MHV68 M2 protein in mediating the suppressive effect of acute MHV68 infection on the generation of humoral immunity to a secondary malaria infection. This work demonstrates that gammaherpesvirus infections can suppress the generation of an effective anti-malaria immune response and suggests that acute EBV infection should be investigated as a risk factor for the development of severe malaria in young children.

## Introduction

Nearly 1 million individuals die annually as a result of severe malaria, largely children under the age of 5 [1]. In regions that are endemic for *Plasmodium falciparum* transmission, mathematical modeling data suggests that immunity to severe non-cerebral malaria requiring hospitalization in children may be attained after 1–2 infections [2]. However, it is not fully understood why some children are unable to acquire immunity to severe lethal disease. Multiple factors may account for this (reviewed in [3,4]) and the presence of co-infecting pathogens in the host could be one such factor. In Sub-Saharan Africa, infants are often co-infected with Epstein-Barr virus (EBV), a gammaherpesvirus that infects B cells and maintains latency throughout the lifetime of the host [5]. Children are often seropositive to EBV by the age of 6 months in this region of the world [6] and it is well established that children infected with EBV living in areas endemic for transmission of *P*. *falciparum* have increased chances of developing endemic Burkitt’s Lymphoma (eBL). eBL is the most lethal of childhood cancers in equatorial Africa, with the highest prevalence in children aged 5–9 years old. eBL is characterized by a c-myc translocation that results in over-expression of the oncogene (reviewed in [7]). It is postulated that repeated infections with *P*. *falciparum* results in a weakened anti-viral CD8 T-cell response that allows for the outgrowth of transformed B cells [8–11].

Despite the compelling evidence indicating a role for *P*. *falciparum* in modulating the immune responses that control EBV infection, little is known regarding the impact of acute EBV infection on the development and functionality of the immune responses that control *P*. *falciparum* infection. It is well appreciated that the humoral response is protective during *Plasmodium* infection. Passive immunization of children in The Gambia [12] and adults in Thailand [13] with *P*. *falciparum* hyper-immune serum from adult donors living in Sub-Saharan Africa allowed for control of peripheral parasitemia. Additionally, numerous studies in humans have identified a role for increased breadth and diversity in the anti-*Plasmodium* humoral response that provides a protective advantage during clinical malaria [14–17]. Although acute EBV infection is generally asymptomatic in young children [18], virus-induced humoral immune deficiencies have been observed in one case of co-infection with a secondary pathogen [19] and in young adults experiencing a primary EBV infection and manifesting symptoms of Infectious Mononucleosis (IM) [20,21]. Although there are few reports of this phenomenon, these documented cases provide key evidence of the ability of EBV to suppress humoral responses during the acute phase of infection. This data, combined with the known role of antibody in resolution of *P*. *falciparum* parasitemia (refs), suggests that overlapping acute EBV infection could suppress anti-malarial humoral responses in some children and thus be a contributing factor in the development of severe malarial disease.

Acute murine gammaherpesvirus 68 (MHV68) infections of mice, like acute EBV infections in humans, can induce a transient immune suppression of the humoral response during secondary antigenic challenge [22]. Using MHV68 as a model for acute EBV infection, we have investigated whether gammaherpesvirus infection can suppress the humoral immune response to a secondary malarial infection. We have used the well-established non-lethal murine models of malaria infection *P*. *yoelii* XNL and *P*. *chabaudi* AS, and determined that acute gammaherpesvirus infection can suppress the anti-malarial humoral response during co-infection with either of these *Plasmodium* infections. This suppression results in loss of control of peripheral parasitemia in *P*. *yoelii* XNL, but not *P*. *chabaudi* AS, infection and transforms the non-lethal infection into a lethal one. This is in agreement with the course of infection in B cell deficient μMT mice where *P*. *chabaudi*, but not *P*. *yoelii*, parasitemia is controlled [23]. The reduced anti-malarial antibody response during co-infection was accompanied with a virus induced failure to maintain the T follicular helper cells subset in the spleen. As such, loss of this critical T helper cell in the germinal center follicle resulted in loss of germinal center B cells and a failure to develop sufficient plasma cells to produce anti-malarial antibody.

We have identified that the MHV68-derived latency associated protein M2 is essential for the failure of co-infected animals to mount anti-malarial humoral responses and that this effect in the mouse model lasted up to 30 days. This identifies the acute phase of infection as necessary for virus-mediated immune suppression of the humoral response. In terms of EBV infection in humans, one case study identified the asymptomatic acute phase of EBV infection to induce immune suppression that can last up to 4 weeks [19]. This potentially gives a 4 week time window of humoral suppression that could substantially influence the outcome of a *P*. *falciparum* infection. As such, our data provides novel and compelling evidence for a need to evaluate primary acute EBV infections as a potential risk factor in the development of non-cerebral severe malaria.

## Results

### Acute MHV68 infection impairs the development of malaria specific antibody responses

**Fig 1:**
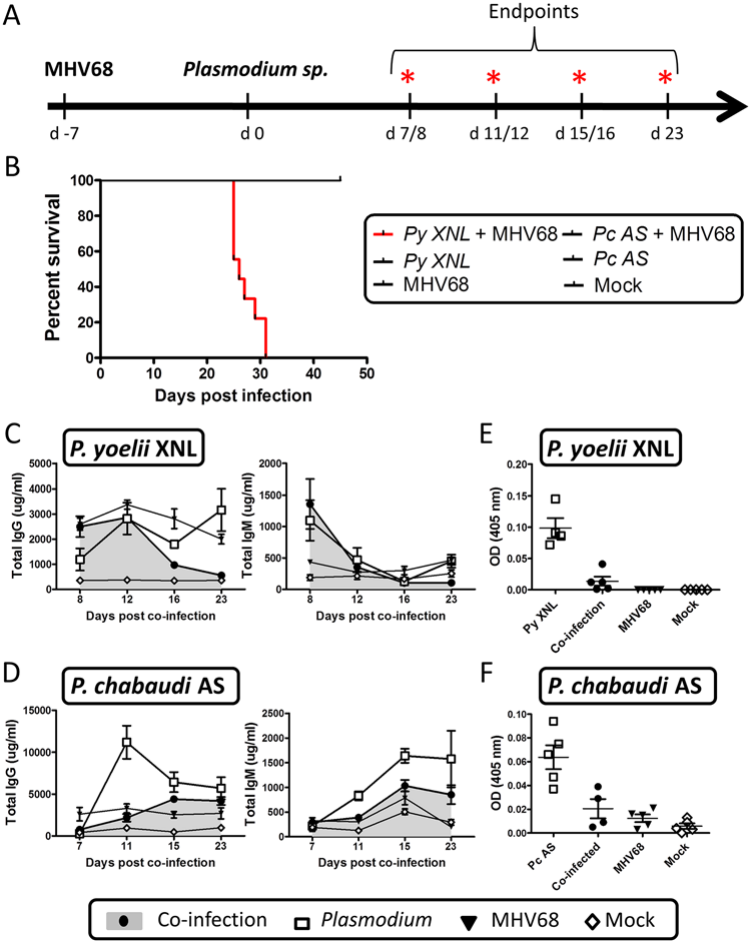
MHV68 co-infection with the non-lethal *P*. *yoelii* XNL in C57BL/6 results in lethal malarial disease and suppressed *Plasmodium* specific IgG response. (A) Timeline of infection. 6–8 week old C57BL/6 mice were infected with 1000 PFU of MHV68 on day -7 followed by infection with 10^5^pRBCs of non-lethal *P*. *yoelii* XNL or *P*. *chabaudi* AS. Infections consisted of 5 experimental groups: MHV68 + *Plasmodium*, *Plasmodium*, MHV68 or mock infected. Each experimental group consisted of n = 5 and was repeated twice. Animals were sacrificed at days 8, 12, 16 and 23 post *P*. *yoelii* XNL infection or day 7, 11, 15 and 23 post *P*. *chabaudi* AS infection for collection of spleen, lung and blood. (B) Survival analysis of animals co-infected with MHV68 and *P*. *yoelii* XNL or *P*. *chabaudi* AS. Total IgG and IgM levels in serum in (C) *P*. *yoelii* XNL (Day 23 IgG—*P*. *yoelii* vs co-infected: p<0.05 Mann Whitney U-test) or (D) *P*. *chabaudi* AS co-infection model (Day 11 IgG—*P*. *chabaudi* vs co-infected: p<0.05 Mann Whitney U-test). Parasite specific IgG levels in serum during (E) *P*. *yoelii* XNL (day 23 post infection, *P*. *yoelii* vs co-infected: p<0.05 Mann Whitney U-test) or (F) *P*. *chabaudi* AS (day 11 post infection, *P*. *chabaudi* vs co-infected: p<0.05 Mann Whitney U-test) co-infection.

The humoral response is generally considered to be a critical effector mechanism for controlling peripheral parasitemia in both human and mouse malaria infection [23]. To understand the impact of acute MHV68 infection on the humoral response to a *Plasmodium* infection, we infected C57BL/6 mice with 1000 PFU of MHV68 intra-nasally (IN) on day -7 and 10^5^ parasitized red blood cells (pRBCs) of *P*. *yoelii* XNL or *P*. *chabaudi* AS intra-peritoneally (IP) on day 0 (Fig 1A). Single infection with either of the *Plasmodium* species was non-lethal but, in the context of an MHV68 infected mouse, *P*. *yoelii* XNL, but not *P*. *chabaudi* AS, caused 100% lethality (Fig 1B). This corroborates a previous observation by Haque et al. who also observed lethality during MHV68 and *P*. *yoelii* XNL co-infection [24]. Knowing the importance of a robust humoral response in protection during *Plasmodium* infection [12,13], we hypothesized that MHV68 may impair the generation of an effective antibody response to control *P*. *yoelii* XNL parasitemia. Total IgM levels were reduced in co-infected animals relative to singly infected animals in *P*. *yoelii* XNL co-infection at day 23 post malaria infection (Mann Whitney-U test p<0.05) and at days 11 and 15 post malaria infection in *P*. *chabaudi* AS co-infection (both Mann Whitney-U test p<0.05) (Fig 1B and 1C). Total IgG levels were similarly affected and reduced at day 23 post malaria infection in *P*. *yoelii* XNL co-infected animals and at day 11 post malaria infection in *P*. *chabaudi* AS co-infected animals (both Mann Whitney U-test p<0.05; Fig 1B and 1D). This reduction in total IgG was mirrored in parasite-reactive IgG in both co-infection models compared with the relevant singly-infected animals (both Mann Whitney U-test p<0.05; Fig 1E and 1F). This observation shows that MHV68 acute infection can suppress the humoral response to malaria infection in mice. In one of the mouse malaria models tested (*P*. *yoelii* XNL), this suppression is correlated with the transformation of a non-lethal malaria infection into a lethal one (Fig 1B). This observation prompted us to evaluate how suppression of the anti-malaria humoral response impacts the control of peripheral parasitemia and to investigate why the acute phase of MHV68 co-infection impacted the virulence of *P*. *yoelii* XNL, but not *P*. *chabaudi* AS infection.

### Acute MHV68 co-infection leads to loss of control of *P*. *yoelii* XNL, but not *P*. *chabaudi* AS parasitemia

**Fig 2:**
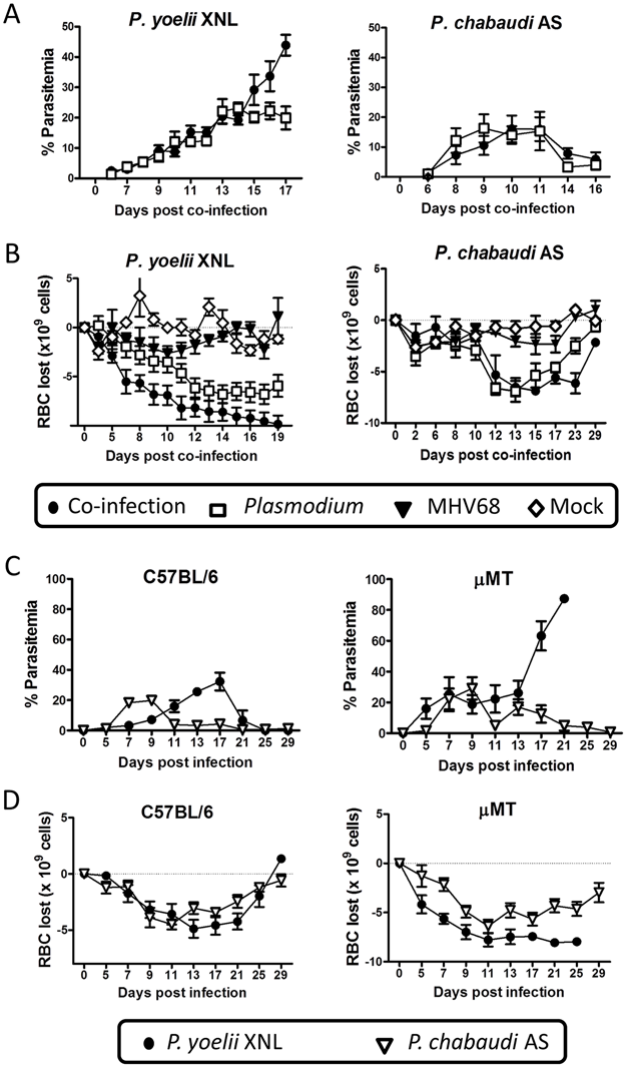
*P*. *yoelii* XNL requires *Plasmodium* specific IgG response to clear primary peak of parasitemia. (A) Percent parasitemia in the periphery during *P*. *yoelii* XNL (p<0.05; area under curve, Mann Whitney U-test) or *P*. *chabaudi* AS co-infection models (p>0.05; area under the curve, Mann Whitney U-test). (B) Anemia during *P*. *yoelii* XNL (p>0.05; area over curve, Mann Whitney U-test, *P*. *yoelii* vs. co-infected) or *P*. *chabaudi* AS co-infection (p>0.05; area over curve, Mann Whitney U-test, *P*. *chabaudi* vs. co-infected). (C) Percent parasitemia in periphery during infection of single *P*. *yoelii* XNL or *P*. *chabaudi* AS in C57BL/6 or μMT (B cell-deficient) mice. (D) Anemia during infection of single *P*. *yoelii* XNL or *P*. *chabaudi* AS in C57BL/6 or μMT mice.

To extend the above observations, we evaluated the impact of an acute MHV68 infection on clearance of the primary peak of parasitemia during secondary challenge with *Plasmodium*. During the initial stages of malaria infection, MHV68 and *P*. *yoelii* XNL co-infected animals had comparable peripheral parasitemia when compared with *P*. *yoelii* XNL singly infected animals (Fig 2A). However, by day 17 post-infection, singly infected animals began to control peripheral parasitemia while co-infected animals were unable to do so (Fig 2A; Mann Whitney-U test p<0.05). There was a trend for co-infected animals to have more severe malarial anemia during *P*. *yoelii* XNL and MHV68 co-infection compared to *P*. *yoelii* XNL singly infected animals, but this did not reach statistical significance (Fig 2B; Mann Whitney-U test on the area above the curve p = 0.056). There was no difference in *P*. *chabaudi* AS parasitemia or anemia in singly infected or MHV68 co-infected groups (Mann Whitney-U test on the area under or above the curve respectively p>0.05) (Fig 2A and 2B).

It is possible that elevated persistent replication of MHV68 in the lungs of co-infected mice could contribe to the lethality of *P*. *yoelii* XNL infection. However, although we observed that levels of preformed virus were significantly higher in the co-infected animals compared to singly infected animals (Mann Whitney-U test on area under the curve p <0.05 at day 23 post *P*. *yoelii* XNL co-infection and day 15 post *P*. *chabaudi* AS co-infection MHV68 infection; S1 Fig, panel A), we also observed a similar increase in viral titer in the lungs of *P*. *chabaudi* AS and MHV68 co-infected animals which was not lethal (S1 Fig, panel B). The elevated persistent viral replication in the lungs of co-infected animals correlated to a decrease in virus-specific IgG in co-infected mice compared to mice infected with MHV68 alone (S1 Fig, panels C & D). Notably, the virus specific antibody response is critical in long term control of viral replication [25]. Assessment of lung tissue at day 23 post co-infection with *P*. *yoelii* XNL and MHV68 indicated increased Type II hyperplasia, which is indicative of interstitial pneumonia (S1 Fig, panel E) and hemosiderin deposition, as compared to animals singly infected with MHV68. In contrast, single MHV68 infection caused greater levels of inflammation in the lung as defined by larger numbers of histiocytes (macrophages and dendritic cells) in the lung tissue (S1 Fig, panel E). Animals co-infected with *P*. *chabaudi* AS and MHV68 showed little to no obvious lung tissue damage as compared to single MHV68 infection (S1 Fig, panel E). Thus, at this point we cannot rule out the possibility that increased persistent replication of MHV68 contributes to the lethality of P. yoelii in co-infected mice.

Based on previously published work [23,26], we hypothesized that the reason why the abolishment of the anti-malarial humoral immunity is lethal for *P*. *yoelii* XNL, but not *P*. *chabaudi* AS infected mice, is because each infection has a differential requirement for a parasite specific antibody response to control the primary peak of parasitemia. To test this hypothesis, we compared the course of infection for both species of rodent malaria in μMT (B cell-deficient) and C57BL/6 mice. We observed that, in the absence of B cells, *P*. *chabaudi* AS infected animals could control the primary peak of parasitemia, whereas, *P*. *yoelii* XNL infected animals developed fulminant parasitemia (Fig 2C). This was also mirrored in the development of a more severe SMA in *P*. *yoelii* XNL co-infected animals (Fig 2D). This data supports the hypothesis that suppression of the anti-*Plasmodium* humoral response in MHV68 co-infected animals is a key factor in why MHV68 co-infection alters the lethality of *P*. *yoelii* XNL, but not *P*. *chabaudi* AS, malaria infection.

### MHV68 impairs the formation of plasma cells in response to secondary malaria infection

**Fig 3:**
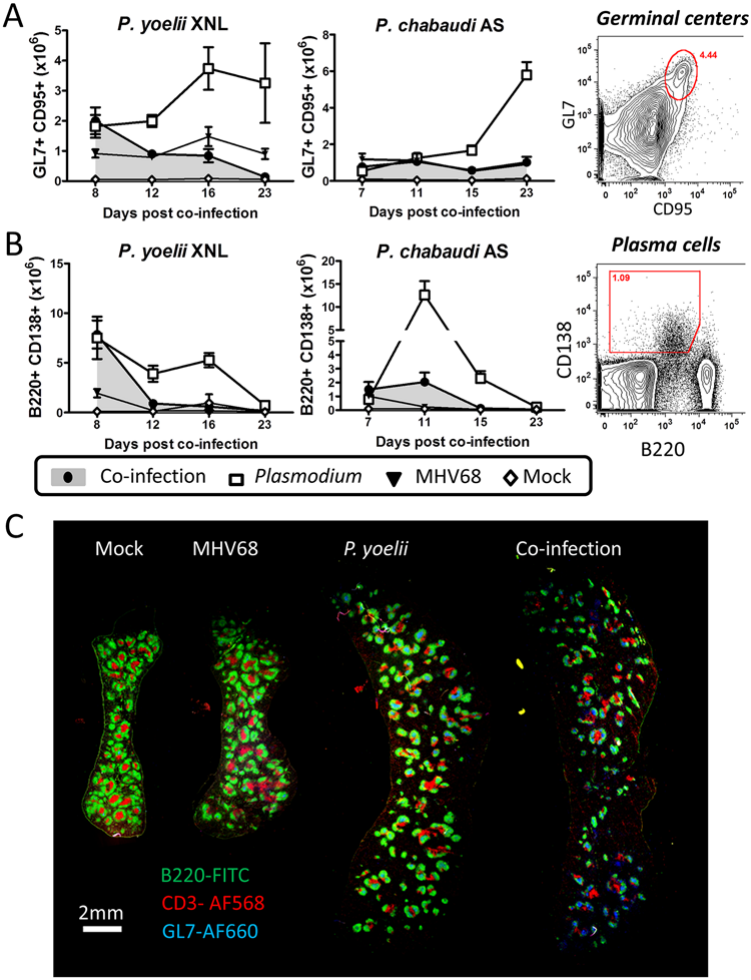
MHV68 suppresses splenic B cell responses during co-infection with *Plasmodium*. The timeline and experimental set up was identical to that shown in Fig 1A. (A) Absolute numbers of splenic GC B cell populations (B220+ GL7+ CD95+) during *P*. *yoelii* XNL and *P*. *chabaudi* AS co-infection models with representative gating strategy (Day 12 post *P*. *yoelii* or Day 15 post *P*. *chabaudi*; *Plasmodium* vs. co-infected, p<0.05, Mann Whitney U-test). (B) Absolute numbers of splenic plasma cell populations (CD3- B220int CD138+) during *P*. *yoelii* XNL AND *P*. *chabaudi* AS co-infection models with representative gating strategy (Day 12 post *P*. *yoelii* or Day 11 post *P*. *chabaudi*; *Plasmodium* vs. co-infected, p<0.05, Mann Whitney U-test). (C) Spleen section for mock infected, MHV68 infected, *P*. *yoelii* XNL infected and MHV68 and *P*. *yoelii* XNL co-infected animals at day 8 post infection with *P*. *yoelii* XNL (or day 15 post-infection with MHV68). Green: B220-FITC (B cells), Blue: GL7-AF660 (Germinal center B cells) and Red: CD3-AF568 (T cells).

We hypothesized that the impairment of the anti-malarial humoral response in MHV68 infected animals was due to a defect in the generation or function of plasma cells upon infection with malaria. We assessed the populations of plasma cells and germinal center (GC) B cells (a precursor of memory and plasma cells) in the spleen at different times post-infection with malaria. Mice that were co-infected with MHV68 and malaria had a comparable number of GC B cells compared to singly infected animals at day 7–8 post infection with malaria (S2 and S3 Figs; co-infected compared with singly infected animals Mann Whitney-U test p>0.05 in both models). However, by day 12 post-infection with *P*. *yoelii* XNL or day 15 post-infection with *P*. *chabaudi* AS, GC B cell numbers were significantly reduced as compared to a single *Plasmodium* infection (both Mann Whitney-U test p<0.05). At day 8 post co-infection, the GC B cells present were located in T cell-containing germinal centers in representative *P*. *yoelii* XNL singly infected and MHV68 co-infected animals (Fig 3C), suggesting that the defect was in the maintenance of the germinal center rather than a follicular structural defect. The impaired GC response correlated with greatly reduced numbers of plasma cells by day 11/12 post-infection with malaria in MHV68 co-infected animals compared with malaria singly infected animals (Mann Whitney-U test p<0.05 in both cases). This observation suggests that the defect in anti-malarial antibody responses to the *Plasmodium* infection in MHV68 co-infected animals is likely due to a defect in the generation and/or maintenance of GC B cells.

### Impaired germinal center maintenance is correlated with reduced Tfh survival

Since germinal center formation and maintenance is dependent on CD4+ T follicular helper cells (Tfh) (reviewed in [27]), we hypothesized that GC B cell numbers may not be sustained if there are detrimental changes in the splenic Tfh population. One notable observation from the representative spleen sections shown in Fig 3C is that the day 8 co-infected mouse appears to have a reduced number of CD3+ T cells within the germinal center follicles compared to the day 8 *P*. *yoelii* XNL singly infected mouse (Fig 3C). Thus, although levels of GC B cells are comparable to a single *P*. *yoelii* XNL infection at this early time point (Fig 3A), a reduction in CD3+ T cells, which would include the Tfh subset, may explain the subsequent decay of the splenic GC population. As such, we next evaluated the T cell repertoire in the spleen that is required for germinal center formation and survival.

**Fig 4:**
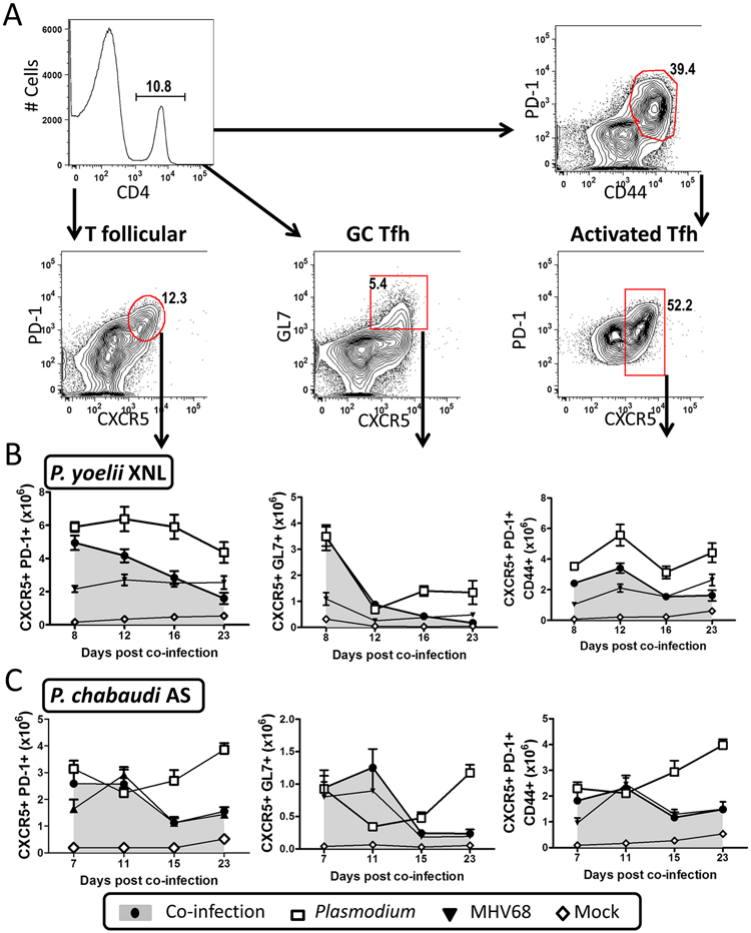
MHV68 and *Plasmodium* co-infection results in defective splenic T follicular helper (Tfh) response. The timeline and experimental set up was identical to that shown in Fig 1A. (A) Representative flow plots for gating strategies used to define the global Tfh population (CD4+ PD-1+ CXCR5+), germinal center Tfh (CD4+ GL7+ CXCR5+) and activated/antigen specific Tfh (CD4+ CD44+ PD-1+ CXCR5+). (B) Absolute values for all three Tfh subsets are plotted for the *P*. *yoelii* XNL (Day 23, all Tfh subsets, *P*. *yoelii* vs. co-infected, p<0.05 Mann Whitney U-test) or (C) *P*. *chabaudi* co-infection models at multiple time points (Day 23, all Tfh subsets, *P*. *chabaudi* vs. co-infected, p<0.05 Mann Whitney U-test).

We analyzed how total Tfh cells (CD4+ CXCR5+ PD-1+), activated/antigen specific Tfh (CD4+ PD-1+ CD44hi CXCR5+) and germinal center Tfh (GL7+ CXCR5+) cells (Fig 4A) changed over time in the spleen. It was evident that by 23 days post-malaria infection there were defects in the maintenance of all three Tfh subsets in co-infected animals when compared to *P*. *yoelii* XNL singly infected animals (Fig 4B; Mann Whitney-U test p<0.05 in all cases). This was mirrored in the MHV68 and *P*. *chabaudi* co-infected animals (Fig 4C). The MHV68 and *P*. *yoelii* XNL co-infected animals displayed defects in the total and activated Tfh subsets as early as 12 days post co-infection (Fig 4B; Mann Whitney-U test p<0.05 in all cases) indicating that MHV68 co-infected animals are capable of generating Tfh responses within the first week after co-infection, but they failed to maintain this cellular subset. The MHV68 induced defect in the Tfh population by day 12 post *P*. *yoelii* XNL co-infection (Fig 4B) also corresponds to the time point at which the GC B cell population begins to decline (Fig 3A). This also applies to the decrease in the Tfh population by day 15 post *P*. *chabaudi* AS challenge (Fig 4C) in MHV68 co-infected animals compared with *P*. *chabaudi* AS singly-infected animals and the corresponding reduction in GC B cell numbers (Fig 3A). This correlation supports the hypothesis that the failure to maintain the population of GC B cells in MHV68 co-infected mice is correlated with a failure to maintain a Tfh cell population.

The defective anti-malarial humoral response induced by an acute pre-existing MHV68 infection may have been a result of alteration of other T cell subsets known to be involved in generating an antibody response, or in the control of parasitemia during malaria infection. To address this, we enumerated numbers of regulatory T cells (Tregs) that can negatively regulate the Tfh response [28] and CD4+T cells that co-express IFN-γ and IL-10 which play an important role in the control of *P*. *yoelii* parasitemia [29]. Acute MHV68 co-infection did not lead to an increase in these subsets in response to malaria infection (S3 and S4 Figs). In fact, Treg numbers were significantly decreased by MHV68 co-infection at day 15/16 post-infection with malaria in both models (S3 Fig, panel B; Mann Whitney-U test p<0.05 in both cases), which we reasoned should enhance, rather than suppress the Tfh response [28], making it an unlikely explanation for suppression of anti-malarial humoral immunity in MHV68 co-infected mice. To further assess the consequences of Tfh deficiency on *P*. *yoelii* XNL compared with *P*. *chabaudi* AS malaria infection, we infected IL-21R-/- mice which can generate comparable levels of Tfh cells and germinal center responses during the early stages of an LCMV infection (around 15 days), but fail to maintain both Tfh and germinal center responses after 2 weeks of infection [30], recapitulating the immunological phenotype of MHV68 and malaria co-infected animals. Similar to μMT mice, *P*. *yoelii* XNL infection of IL21R-/- mice was lethal and this was associated with impaired control of parasitemia and a concomitant increase in the severity of SMA (S5 Fig, panels A and B). In contrast, IL-21R deficiency did not affect the kinetics of a *P*. *chabaudi* AS single infection (S5 Fig). Collectively, this data supports the hypothesis that the failure of MHV68 co-infected animals to maintain the Tfh cellular subset in the spleen is associated with a defective humoral response against a secondary malaria infection, which in the case of a *P*. *yoelii* XNL infection, results in lethality.

### Acute, but not latent MHV68 infection, is required for exacerbated malarial disease during co-infection

**Fig 5:**
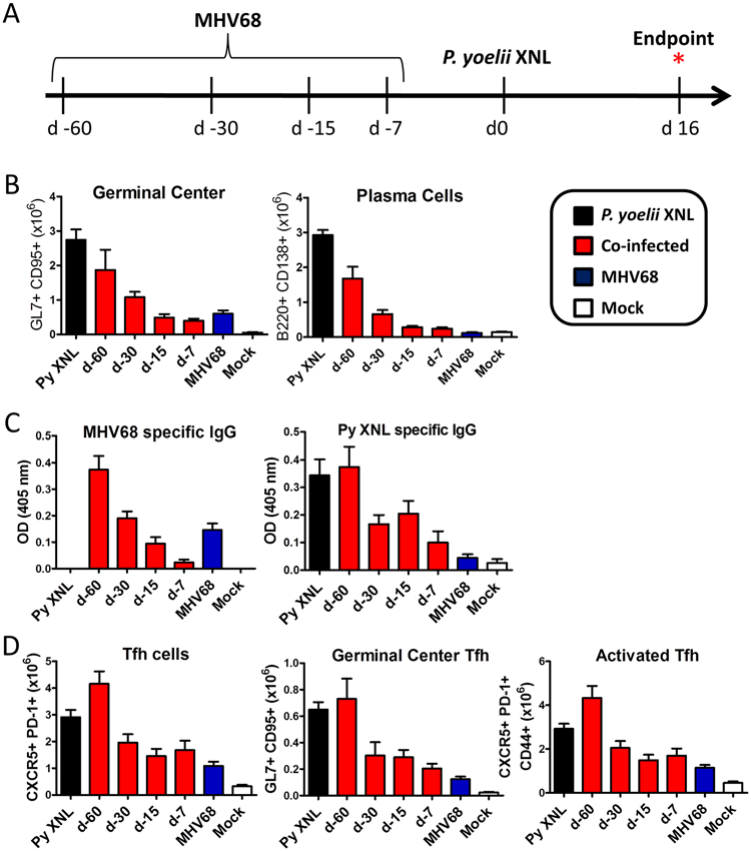
Acute, but not latent, MHV68 infection results in suppressed humoral response. (A) Timeline of infection. C57BL/6 mice were infected with 1000 PFU of MHV68 IN at day -60, -30, -15 or -7 and challenged with 10^5^ pRBCs on day 0. Absolute number of (B) splenic GC B cell (B220+ GL7+ CD95+) and plasma cell (CD3- B220int CD138+) populations at day 16 post *P*. *yoelii* XNL infection (For GC and PC: Day -7 and Day -15 co-infected vs. *P*. *yoelii*, Kruskal Wallis p<0.05; Dunn’s pairwise comparison test p<0.05/ Day -30 co-infected vs. *P*. *yoelii*, Kruskal Wallis p<0.05; Dunn’s pairwise comparison test p>0.05). (C) MHV68 and *P*. *yoelii* XNL specific IgG responses at day 16 post *P*. *yoelii* XNL infection (Day -7 and Day -15 co-infected vs. *P*. *yoelii*, Kruskal Wallis p<0.05; Dunn’s pairwise comparison test p<0.05/ Day -30 co-infected vs. *P*. *yoelii*, Kruskal Wallis p<0.05; Dunn’s pairwise comparison test p>0.05). (D) Global Tfh population (CD4+ PD-1+ CXCR5+), germinal center Tfh (CD4+ GL7+ CXCR5+) and activated/antigen specific Tfh (CD4+ CD44+ PD-1+ CXCR5+) in the spleen at day 16 post *P*. *yoelii* XNL infection.

Given that all children in an endemic area would likely be co-infected with EBV and malaria by the time they are 2 years of age, we hypothesized that gammaherpesvirus induced suppression of the establishment of an anti-malarial humoral responses would depend on the timing of co-infection. Gammaherpesvirus infections, such as EBV and MHV68, can be divided into 2 distinct phases: a lytic phase in which there is acute virus replication and dissemination, followed by the establishment of viral latency in B cells and some other cell types. Latent infection consists of a quiescent phase of viral gene expression, but still results in an underlying inflammatory response [5]. Therefore we investigated whether latent infection as well as acute infection resulted in a defect in the development of the anti-malarial humoral response during co-infection. C57BL/6 mice were infected with MHV68 at 60 (latency), 30 (latency), 15 (late lytic- acute) or 7 (early lytic- acute) days prior to being co-infected with *P*. *yoelii* XNL (Fig 5A). We measured the number of GC B cells and plasma cells (Fig 5B), the levels of circulating *P*. *yoelii* XNL specific IgG responses (Fig 5C) and the numbers of Tfh cellular subsets in the spleen (Fig 5D).

As expected, animals infected at day -7 prior to *P*. *yoelii* XNL infection showed a marked reduction in all of these parameters compared to mice singly infected with *P*. *yoelii* XNL (Kruskal-Wallis p<0.05; Dunn’s pairwise comparison p<0.05 in all cases). This pattern was repeated in animals infected with MHV68 at 15 days prior to *P*. *yoelii* XNL infection (Kruskal-Wallis p<0.05; Dunn’s pairwise comparison p<0.05 in all cases) and there was a trend towards this pattern in mice infected with MHV68 for 30 days prior to *P*. *yoelii* XNL infection that did not reach significance (Kruskal-Wallis p<0.05; Dunn’s pairwise comparison p>0.05 in all cases). However, it is clear that the suppressive effects of an acute MHV68 infection were not present in mice that were latently infected with MHV68 60 days prior to infection with *P*. *yoelii* XNL. This data suggests that an established latent MHV68 infection does not suppress the generation of the humoral immune response to an incoming malaria infection in mice.

The mechanism by which acute MHV68 infection can suppress the generation of a humoral response to malaria during co-infection is unclear, but GC B cells from animals with MHV68 co-infection 15 or 7 days prior to *P*. *yoelii* XNL infection had increased expression of PD-L1 (S6 Fig), a ligand for PD-1 that negatively regulates Tfh expansion [31], relative to *P*. *yoelii* XNL singly-infected animals (Kruskal-Wallis p<0.05; Dunn’s pairwise comparison p>0.05 in both cases). Thus, one possibility is that virus induced PD-L1 expression on GC B cells may contribute to the loss of Tfh functionality during co-infection.

### The MHV68 M2 gene product plays a role in suppression of anti-parasitic humoral responses

**Fig 6:**
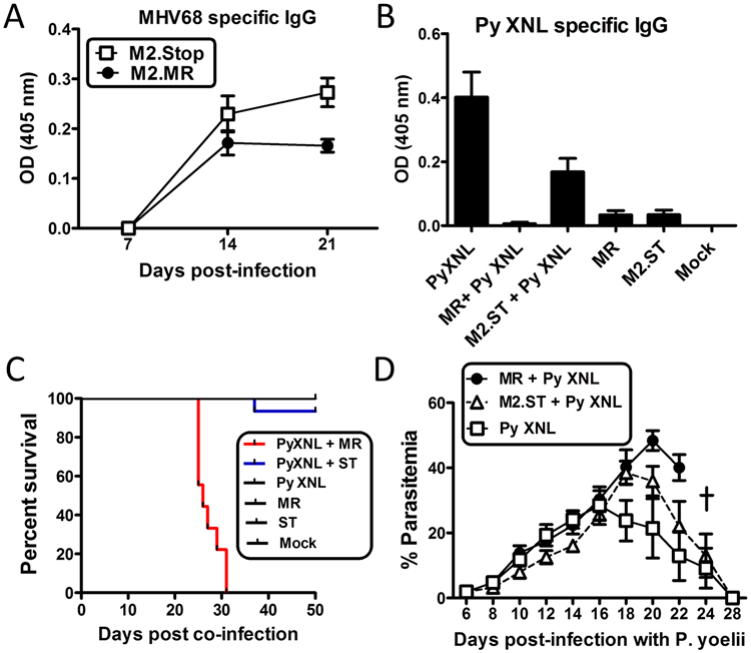
The MHV68 M2 gene product is necessary for virus mediated humoral suppression and lethality during *Plasmodium* co-infection. (A) MHV68 specific IgG titers from serum of animals infected with the MR (M2.Marker Rescue) or M2.Stop (ST, M2-null) viruses. Serum was collected and analyzed on days 7, 14 and 21 post infection with either virus (n = 10/ virus) (Day 21, MR vs. M2.Stop, Kruskal Wallis p<0.05; Dunn’s pairwise comparison test p<0.05). (B) *P*. *yoelii* XNL specific IgG response during *P*. *yoelii* XNL co-infection with either the M2.MR or M2.Stop virus. Serum was collected at day 20 post infection with *P*. *yoelii* XNL (WT + *P*. *yoelii* co-infected vs. *P*. *yoelii*, Kruskal Wallis p<0.05; Dunn’s pairwise comparison test p<0.05/ WT + *P*. *yoelii* co-infected vs. M2.Stop + *P*. *yoelii*, Kruskal Wallis p<0.05; Dunn’s pairwise comparison test p>0.05). (C) Survival curve during *P*. *yoelii* XNL co-infection with either the M2.MR or M2.Stop virus. Note: data representing *P*. *yoelii* XNL + MHV68 co-infection is the identical data set to that in Fig 1B. It was added in panel C for comparative purposes. (D) % parasitemia in the periphery during *P*. *yoelii* XNL, *P*. *yoelii* XNL +MR and *P*. *yoelii* XNL + M2.Stop infection.

The data presented above clearly points to a suppressed humoral response as being a critical mediator of lethality during *P*. *yoelii* XNL co-infection with MHV68. As such, we hypothesized that if we could restore the parasite specific humoral response, we could rescue mice from lethality caused by an MHV68 and *P*. *yoelii* XNL co-infection. It has previously been shown that the M2 gene product of MHV68 can induce significant levels of IL-10 production from B cells and modulate the surface phenotype of infected B cells [32,33]. IL-10 is known to have multiple immunomodulatory roles, one of which is to negatively regulate T cell responses [34,35]. We hypothesized that one reason Tfh cells did not function correctly could be due to M2-induced suppression, a hypothesis supported by published work showing that in the absence of M2, mice are able to mount enhanced virus specific CD8+ T cell responses [33]. Given that the downstream effect of MHV68 induced immunosuppression was a result of impaired anti-malarial antibody responses, we initially asked whether levels of virus specific IgG responses were enhanced in the absence of M2 expression. To avoid a known defect in the establishment of splenic infection following intranasal inoculation of M2-deficient MHV68 mutants [36]we opted to infect mice with the same dose of virus (1000 PFU) as used in the previous experiments, but via the intraperitoneal route—a route and dose of virus which allow the M2-deficient mutant to efficiently infect the spleen [36]. As a proper control, the marker rescue virus (i.e, a recombinant MHV68 in which the genetic mutation introduced into the M2 null mutant was restored to the wild type virus sequence) [37], was also administered via the IP route. Notably, we have extensively compared IN versus IP MHV68 co-infection with *P*. *yoelii* and found there to be no difference in outcome. M2.Stop (M2 null virus; M2.St) infection in a C57BL/6 mouse induced a nearly 2-fold higher MHV68 specific IgG response as compared to infection with the marker rescue control virus (M2.MR; MR) (Fig 6A; day 21 post-infection Mann Whitney-U test p<0.05). It is important to note that day 21 post-MHV68 infection in this experiment corresponds to day 14 post-co-infection with *P*. *yoelii* XNL. The time point at which the virus specific humoral response is suppressed overlaps with the timing at which parasite specific IgG responses become severely compromised during co-infection (Fig 1C and 1E). This observation suggests that M2 may be mediating the virus-induced suppression of anti-malarial humoral immune responses.

To evaluate whether loss of M2 expression could alleviate the MHV68 induced suppression of the anti-malarial humoral response, we infected mice with either the M2 null mutant (M2.Stop) or the marker rescue virus control (MR) 7 days prior to challenge with *P*. *yoelii* XNL. While co-infection with the MR virus suppressed the generation of anti-*P*. *yoelii* XNL specific IgG (Kruskal Wallis p<0.05; Dunn’s pairwise comparison test p<0.05), mice that were co-infected with M2.Stop showed a 28-fold increase in *P*. *yoelii* XNL specific IgG at day 23 post co-infection relative to the lethal MHV68 and *P*. *yoelii* XNL co-infection model (Kruskal Wallis p<0.05; Dunn’s pairwise comparison test p>0.05) (Fig 6B). Importantly, 94% of mice co-infected with M2.Stop and *P*. *yoelii* XNL survived (1 out of 15 mice died) (Fig 6C) compared to 100% lethality in mice co-infected with MR and *P*. *yoelii* XNL. Consistent with this observation, circulating parasitemia was not detectable by microscopy at day 28 post co-infection in the M2.Stop and *P*. *yoelii* XNL co-infected mice (Fig 6D). This data clearly establishes a strong link between MHV68 mediated suppression of the humoral response to *P*. *yoelii* XNL and survival. In addition, it argues for a role of the MHV68 M2 protein in mediating the suppression of the anti-malarial humoral response in MHV68 co-infected mice.

## Discussion

The risk factors postulated to contribute to disease severity in young children infected with malaria are numerous and include co-infection with other pathogens [4]. Many children in Equatorial African countries are seropositive for EBV by the age of 6 months as protective maternal antibodies wane [6,38], indicating that primary infection with EBV coincides with the time at which the risk of developing severe malaria is greatest [6,18]. Acute infection with EBV is asymptomatic in young children [18] and results in a latent infection that persists for the life time of the host. Children who experience recurrent infection with *P*. *falciparum* while latent for EBV show an impairment in virus specific CD8 T cell responses [8,39–42] which contributes to an increased risk of developing eBL [reviewed in [7]]. While the impact of repeated *P*. *falciparum* infections can abate an EBV-specific adaptive immune response, little has been known regarding the impact of acute EBV infection on severe malarial disease during childhood.

The risk of co-infection with *P*. *falciparum* before the age of 1 is extremely high for children living in Sub-Saharan Africa [6,18] and for some children it is likely that their primary infections of EBV and *P*. *falciparum* will overlap. There are several reports that asymptomatic EBV infection can have suppressive effects on the host’s humoral adaptive response. One prominent example involves a case study on a child aged 2 ½ years presenting with a recurrent case of otitis media and pneumonia. It was established that this child exhibited suppressed humoral responses when immunized with bacteriophage ɸX174 or Keyhole limpet hemocyanin (KLH) during an asymptomatic infection with EBV. Increased EBV specific antibody titers correlated with a suppression in secondary humoral responses to unrelated antigens [19]. The same observation has been documented in young adults experiencing a primary EBV infection and manifesting symptoms of Infectious Mononucleosis (IM) [20,21]. Similarly, Holder et al. recently described a role for acute EBV infection in attenuating vaccine specific antibody responses in Gambian children as compared to children who had concurrent CMV infection [43]. These observations were also extended to the marmoset model where Wedderburn et al. demonstrate that acute co-infection of EBV and *P*. *brasilianum* resulted in severe morbidity and death [44]. Collectively, these documented cases provide key evidence of the immune suppressive nature of an acute EBV infection on the development of humoral immunity. More importantly, several studies have shown a correlation between non-cerebral severe disease (particularly severe malarial anemia [SMA]) and attenuated parasite specific antibody responses [14,45].

Here, using well characterized mouse models, we provide evidence that acute gammaherpesvirus infection can suppress the development of humoral immunity to a secondary *Plasmodium* infection in two different non-lethal models of rodent malaria (Fig 1). Interestingly, this defect correlated with a transformation of a non-lethal malaria infection into a lethal one in the case of *P*. *yoelii* XNL co-infection, but had no obvious impact on the pathogenesis of *P*. *chabaudi* AS infection (Fig 2). This result is likely to be due to the differential role of antibody mediated parasite clearance mechanisms in controlling the primary peak of parasitemia in each of these models (Figs 2 and S5) [23,26], although the effects of a primary acute MHV68 infection on other cells types such as macrophages cannot be ruled out. The reason why *P*. *yoelii* XNL infection is more dependent on antibodies for the control of parasitemia than *P*. *chabaudi* AS is unknown, but could be related to the kinetics of infection. In our hands, *P*. *yoelii* XNL parasitemia in intact C57BL/6 mice peaked significantly later than *P*. *chabaudi* AS infection (15.5 ± 1.5 days compared with 8.2 ±0.5 days; Mann Whitney-U test p<0.05). T cells, in particular IFN-γ-producing T cells [46–49], have been implicated in orchestrating the control of peripheral parasitemia in both models, whereas IL-10 producing T cells have been shown to exacerbate *P*. *yoelii* XNL parasitemia [29]. Therefore, it is possible that MHV68 infection resulted in an alteration of T cell phenotypes generated against a secondary malaria infection that impacted the pathogenesis of the infection. However, these splenic CD4+ T cell populations measured at different time points post-infection were not significantly altered (S4 Fig), and IL-10-producing CD4+T cells were less in number during MHV68 and *P*. *yoelii* XNL co-infected animals as compared to *P*. *yoelii* XNL singly infected animals (S4 Fig, panel B), which theoretically should lead to better control of peripheral parasitemia [29]. Furthermore, T cells have been shown to play a critical role in the control of the primary peak of *P*. *chabaudi* AS infection [48,49], yet MHV68 co-infection did not alter the peripheral parasitemia in this model suggesting that the relevant defect lies within the failure to mount an appropriate humoral response.

Despite the differential outcome of the two co-infection models, the impact of acute viral infection on the suppression of the humoral response is a common feature of MHV68 and malaria co-infection in C57BL/6 mice. Our data suggests that co-infected mice have a profound defect in the ability to form antibody producing plasma cells (Fig 3). GC B cells are precursors of plasma cells and are dependent on the T follicular helper subset for development and maintenance (reviewed in [50,51]). Analysis of germinal center formation at day 8 post-infection with *P*. *yoelii* XNL demonstrates that germinal centers can form in the spleens of co-infected mice, but are not maintained (Fig 3). This may be due to a defective ability of GC B cells to communicate with Tfh cells due to elevated expression of the suppressive ligand PD-L1 (S6 Fig). PD-L1 mediates its inhibitory role by ligating the PD-1 (Programmed Death-1) receptor on T cells. Recent studies suggest that the reduction in HIV specific antibody responses during chronic infection is correlated with an up-regulation of PD-L1 expression on germinal center B cells [52]. It is interesting that the possible cause of the Tfh impairment may be associated with a change in the surface phenotype of the GC B cell. This is particularly significant in the case of MHV68 infection, since B cells are the primary cell infected by this virus. However, it is unclear whether MHV68 directly affects the GC B cell, or whether soluble mediators of infection suppress the function of Tfh cells in their ability to support the transformation of GC B cells to plasma cells. Our data does not support a role for suppressive effects of FoxP3+ Tregs (S3 Fig) in mediating this effect since the expansion of Tregs is comparable during co-infection compared to a single *Plasmodium* infection. (S3 Fig, Mann Whitney-U test p<0.05 in both cases).

The suppression of the anti-malarial humoral response is evident during acute, but not latent, MHV68 infection (Fig 5). MHV68 has evolved elaborate immune evasion strategies to survive the potent innate inflammatory responses that it induces during acute infection [53]. One interesting observation previously made in the Speck laboratory noted that loss of M2 expression *in vivo* resulted in a more robust anti-viral CD8 T cell response [33]. M2 is a unique viral protein expressed by MHV68 which shares some functional homology with the LMP1 and LMP2a EBV gene products—which mimic CD40 and BCR signaling, respectively [54,55]. M2 is able to promote signals downstream of the BCR receptor [56,57], induces IL-10 production from B cells [33,37] and promotes differentiation of infected B cells into plasma cells (note that ≤ 1% of B cells are MHV68 infected at the peak of latency) [32]. One notable effect of M2 expression *in vivo* is the dramatic increase of IL-10 levels in the serum of infected mice [33]. IL-10 is an immune-modulatory cytokine which is capable of suppressing T cell activation [35]. M2 mediated reduction of the anti-viral CD8 T cell response likely reflects an evolutionary viral adaptation that allows for the evasion of the immune response, and more importantly, allows for establishment of viral latency. Since the humoral response is dependent on a robust T cell response, we predicted that M2 may also influence the generation of a virus specific IgG response, a critical branch of the adaptive response involved in long term clearance of the virus [25]. Our data shows that in the absence of M2 expression, the development of an anti-viral humoral response was enhanced 2-fold (Fig 6). Additionally, co-infection with M2.Stop mutant virus and *P*. *yoelii* XNL showed a 28-fold increase in the anti-malarial IgG response, which also correlated with survival during co-infection. We compared virus specific IgG levels in mice infected with another unrelated viral mutant that is null for M1 protein expression. Both the M1.Stop and M1.MR viruses showed equivalent levels of virus specific IgG responses over 2 months of infection (S7 Fig), suggesting an M2-specific role in suppressing the virus specific humoral response. This further corroborates data shown by Getahun et al. [22], which demonstrated that infection with M1.Stop or M3.Stop (a viral chemokine) could not alleviate the virus-induced immune suppression. We currently do not understand the mechanism by which M2 is mediating this effect, although we hypothesize that increased IL-10 production from B cells in the splenic follicle may negatively regulate Tfh survival and consequently affect germinal center maintenance and development. Although B cell expansion is beneficial for seeding viral latency and persistence, it is evident that the virus also negatively regulates the production of virus specific IgG responses, a key immune evasion mechanism which would support viral persistence. Although M2 expression is associated with IL-10 production, we cannot rule out the possibility that M2 is mediating its effect in a non-IL-10 dependent manner or that M2-driven IL-10 expression may impact other aspects of the host response that may contribute to the observed lethality of MHV68 and P. yoelii co-infection. However, our novel discovery implicating M2 in mediating the virus induced humoral suppression against secondary parasitic infection is a key observation for future efforts in dissecting the mechanism behind this observed phenotype.

As with every model system, certain limitations exist. Our studies rely on infection of 6–8 week old mice since this age group has been extensively studied. The limited studies in neonatal mice suggest that BALB/c mice, and not C57BL/6 mice, are more susceptible to infection and may develop myocarditis and neurologic disorders [58,59]. It appears that neonate C57BL/6 mice, which is the background used in our studies, do not experience altered immune responses compared to adults. As such, there is little premise to suggest that younger mice would react differently to acute MHV68 infection. However, the impact of co-infection on malarial disease severity in neonate mice has not been explored and is worth pursuing in other studies. Another important aspect worth noting is that the isolated system used to model human co-infection cannot encompass the myriad of factors influencing malarial disease severity in humans. It has been extensively demonstrated that various other viral, bacterial, and helminth co-infections impact malarial disease [60–65]. Additionally, factors such as parasite virulence, nutrition, host health and genetics can also contribute to the variation in malarial disease severity [3,4]. As such, acute EBV infection is unlikely to be the sole contributor in modulating malarial disease. However, the results reported in this manuscript aim to elucidate previously neglected co-infections, such as ubiquitous asymptomatic EBV infection, in altering non-cerebral malarial disease severity. More importantly, multiple human reports indicate that asymptomatic acute EBV infection has the ability to alter the generation of a humoral response during secondary pathogen challenge [19–21,43,44] and demonstrated by us and others [22] during MHV68 acute infection. Collectively, our observations in the mouse model and supporting literature of human studies provide a strong premise for investigating the role of acute EBV in malarial disease. Undoubtedly, detailed longitudinal studies are required in humans to conclusively establish this correlation.

In conclusion, our work provides compelling evidence that acute gammaherpesvirus infection can negatively modulate the humoral immune response to malaria infection. This data provides justification to investigate how EBV infection might impact the development of *P*. *falciparum* humoral immunity in young children living in malaria endemic areas. If found to be a risk factor for developing severe malaria, tackling EBV infection via the development and use of an EBV vaccine or anti-viral therapies in malaria endemic areas, may provide some relief in the development of non-cerebral severe disease during childhood malaria infection.

## Materials and Methods

### Ethics statement

This study was carried out in strict accordance with the recommendations in the Guide for the Care and Use of Laboratory Animals of the National Institutes of Health. The protocol was approved by the Emory University Institutional Animal Care and Use Committee, and in accordance with established guidelines and policies at Emory University School of Medicine (Protocol Number: YER-2002245-031416GN).

### Mice and infections

Female C57BL/6 mice (6–8 weeks) were purchased from Jackson Laboratories. μMT and RAG2-/- mice were purchased from Jackson laboratories and bred in house. IL21R-/- mice were a gift of Dr. Warren J. Leonard and were bred in house. Mice were infected with 1000 Plaque Forming Units (PFU) of MHV68 in DMEM without fetal bovine serum (FBS) either Intra-Nasally (IN) or Intra-Peritoneally (IP). Animals are infected IN with MHV68 throughout the manuscript, unless where otherwise noted. Frozen stabilates of *P*. *yoelii* XNL or *P*. *chabaudi* AS were administered IP in Kreb’s saline with glucose (KSG) into donor mice. After day 7–9 after infection of the donor mouse, experimental mice were infected at a dose of 1 x 10^5^ parasitized Red Blood Cells (pRBCs) via the IP route in KSG. Anemia was measured by counting RBCs from tail blood diluted in KSG using a haemocytometer [66]. Parasitemia was enumerated from Giemsa stained thin blood smears.

### Limiting dilution analysis of viral lung titer and lung tissue histology

The left lung was collected for analysis of viral replication using a limiting dilution analysis as previously described [67]. Briefly, lungs were homogenized in 1ml of complete DMEM media and 1.0 mm Zirconia/Silicon beads (BioSpec products) using a BioSpec mini bead-beater 16. Samples were homogenized 4 times (1 minute homogenization followed by 1 minute rest on ice). Samples were then transferred to a new tube with 0.5 mm Zirconia/Silicon beads and homogenization was repeated as above. Homogenate was then plated on Mouse Embryonic Fibroblasts (MEFs) in 96 well plates in serial 2 fold dilutions, up to 12 dilutions. Plates were incubated for 2 weeks in a low evaporation incubator (5% CO_2_, 37°C) and analyzed for Cytopathic effect (CPE). Results are plotted as percent of wells displaying CPE at each plated dilution. For lung tissue histology, the left lung tissue was collected for lung histology analysis. Whole tissue was fixed in 10% (v/v) Normal Buffered formaldehyde for 24 hours at room temperature. Tissue was then put into 70% ethanol solution until samples were sectioned. Samples were paraffin embedded and prepared for Hematoxylin and Eosin Staining as previously described [68].

### Flow cytometry

Splenocytes were blocked with anti-CD16/32 (BD bioscience). Surface stains were performed in PBS-2% FBS- 1mM EDTA for 20 minutes on ice. Markers used: CD138-PE (BD bioscience), B220-Pacific Blue (Biolegend) or Pacific Orange (Invtirogen), CD95-PE-Cy7 (BD biosciences), GL7-Alexa Fluor 660 or FITC (eBioscience), CD3-PerCP/ Pacific Blue (BD bioscience), CD4-PerCP (BD Bioscience), CD8-Pacific Orange (Invitrogen), IL-10-PE (Biolegend), IFN-γ-APC (ebioscience), IL-2 PE-Cy7 (ebioscience) or FITC (Biolegend), purified anti-CXCR5 (BD biosciences), Biotin-SP-AffiniPure F(ab')2 Fragment Goat Anti-Rat IgG (H+L) (Jackson immunoresearch), Streptavidin-APC (Molecular probe), CD279-PE (Biolegend), CD44-Pacific Blue (Biolegend), CD25-Pacific Blue (Biolegend), FoxP3-APC (ebioscience) and CD19-FITC/PE/PE-Cy7/PerCP/ APC/Pacific Blue (BD bioscience). Intracellular cytokine stains for IL-2, IL-10 and IFN-γ were performed using the BD bioscience Cytofix/Cytoperm staining kit. Cells were stimulated (5 hours, 5% CO_2_, 37°C) in anti-CD3 coated 96 wells tissue culture plates and supplemented with soluble anti-CD28 and Brefeldin A. Intracellular staining for FoxP3 was performed using the eBioscience FoxP3 staining kit. Staining for Tfh cells was performed as previously described [69]. Fixable live dead stains were purchased in FITC from Life Technologies and Zombie Yellow (Pacific Orange) from Biolegend and used according to manufacturer’s guidelines. Stained splenocytes were fixed in 2% formaldehyde prior to analysis. Samples were read on a BD LSRII. Data was analyzed using FACS Diva and FloJo software.

### Blood collection and ELISA

For serum samples, blood was collected by cardiac puncture during terminal bleeds. Blood was allowed to clot at 4°C for 1 hour. Tubes were spun at 4°C at 14,000 rpm for 2 minutes. Serum was transferred to a fresh tube and stored at -80°C. For plasma collection, 100μl of blood was collected from the tail vein into Lithium-heparin coated tubes (BD microtainer). Tubes were spun at 4°C at 10,000 rpm for 10 minutes. Plasma was transferred to a fresh tube and stored at -80°C. ELISA assays were performed as previously described [70]. Briefly, 96 well Nunc ImmunoMaxisorp ELISA plates were coated with 0.5 ug/well of goat anti-mouse IgG or IgM antibody (Southern Biotech) or sucrose gradient purified MHV68 in PBS. Serum was serially diluted (3 fold, beginning at 1:100) and 6 dilutions were plated for each sample. Alkaline Phosphatase conjugated goat anti-mouse IgG or IgM (Southern Biotech) was used as a secondary antibody. For parasite specific ELISAs, mice were infected with 10^5^ pRBC of *P*. *yoelii* XNL or *P*. *chabaudi* AS. Blood was harvested and pooled from infected mice. pRBCs were purified using a Percoll gradient. Purified pRBCs were then cultured for schizont maturation for 4 hours in a shaking 37°C water bath in RPMI media supplemented with 10% FBS, 100ug/ml Streptomycin, 100U/ml Penicillin, L-Glutamine (2 mM), HEPES (6 mM), β-mercaptoethanol (50 μM) and sodium pyruvate (0.5 mM). pRBC schizonts were then spun out of culture and lysed with lysis buffer (50 mM Tris/HCl + 1 mM EDTA + 0.5% SDS). Optical Density (OD) of homogenate was read at 206 nm. Plates were coated with homogenate at an OD of 0.1–0.2. ELISA protocol for the parasite specific response was performed as previously reported [71] Color was developed using p-nitrophenyl phosphate (Sigma) in a diethanolamine substrate buffer. Absorbance at 405 nm was read on a Biotek Synergy HT reader. Data is represented as absorbance at 405 nm.

### Immunofluorescence staining and microscopy

Whole spleen was collected at day 8 (post parasite infection) from animals that were MHV68, *P*. *yoelii* XNL, MHV68 + *P*. *yoelii* XNL or mock infected. The whole spleen was embedded in Tissue-Tek OCT media (Sakura-Finetek) and frozen in chilled Isopentane. 7 μm tissue sections were mounted on slides and allowed to dry at room temperature for 12 hours after which they were frozen at -80°C for long term storage. For staining, slides were allowed to equilibrate to room temperature then rehydrated in PBS for 10 minutes. Sections were stained using B220-FITC (BD bioscience), GL7-AF660 (eBioscience) and a primary purified biotinylated CD3 (eBioscience) with secondary anti-Streptavidin in AF-568 (Life Technologies). Sections were blocked in 5% normal mouse serum in PBS for 20 minutes at room temperature. Primary stains were incubated in block solution for 1 hour and secondary stains for 30 minutes. Sections were washed 3 times with PBS and then mounted with Prolong anti-fade without DAPI (Cell signaling) and #1.5 Fisherbrand microscope slides. Mounted sections were allowed to cure in the dark at room temperature for 24 hours. Fluorescence images were taken on an Olympus Fluoview FV1000 with a 10X 0.3 NA objective and utilizing the multi-area time lapse (MATL) xy-stitching functions. The confocal pinhole was opened to 300 μm to increase the thickness of the optical section facilitating the single plane image. Entire spleen sections required ~100 to 200 images, 1600 x 1600 pixels (~850 x 850 μm) at a zoom of 1.5 and zero overlap. This exceeds the 15000 x 15000 stitching pixel limit of the Fluoview software, and as such a Fiji plugin was written to convert MATL log files for use with the stitching plugin within Fiji [link to http://ici.emory.edu/Resources/plugins.html].

## Supporting Information

S1 Fig Increased viral persistence and reduced virus specific IgG response during MHV68 and *Plasmodium* co-infection. The timeline and experimental set up was identical to that shown in Fig 1A. Limiting dilution analysis of viral lung titers in the (A) *P*. *yoelii* XNL or (B) *P*. *chabaudi* AS co-infection models at multiple times post co-infection. MHV68 specific IgG titers in the serum at day 23 post *Plasmodium* infection in (C) *P*. *yoelii* XNL or (D) *P*. *chabaudi* AS co-infected mice. (E) Hematoxylin and eosin stain of lung tissue sections from animals sacrificed at day 23 post co-infected with MHV68 and either *P*. *yoelii* XNL or *P*. *chabaudi* AS (mock and MHV68 infected lung sections are also shown). Scale bar 100 μm.
(TIF)

S2 Fig Gating strategies for removing doublets and discriminating between and live vs. dead lymphocyte populations in the spleen. Identification of live cells was done using a fixable viability dye (Life Technologies). SSC-A, side scatter area; FSC-A, forward scatter area; FSC-W, forward scatter width; FSC-H, forward scatter height; SSC-W, side scatter width; SSC-H, side scatter height.
(TIF)

S3 Fig MHV68 and *Plasmodium* co-infection does not alter CD4+ Th1 responses in the spleen. The timeline and experimental set up was identical to that shown in Fig 1A. (A) Representative flow panels show gating strategies. Cells were gated on the live population, and CD4+ cells were analyzed for cytokine production. Absolute number of CD4+ T cells producing IL-10, IFN-γ, or both in the (B) *P*. *yoelii* XNL and (C) *P*.*chabaudi* AS co-infection models.
(TIF)

S4 Fig MHV68 and *Plasmodium* co-infection does not alter the CD4+ T regulatory (Tregs) subset in the spleen. The timeline and experimental set up was identical to that shown in Fig 1A. (A) Representative flow plots showing gating strategy for Tregs (CD4+ CD25+ FoxP3+). Absolute numbers of Tregs in the spleen at indicated time points for (B) *P*. *yoelii* and (C) *P*. *chabaudi* co-infection models.
(TIF)

S5 Fig *P*. *yoelii* XNL infection IL-21R-/- mice is lethal. (A) % parasitemia and anemia during *P*. *yoelii* XNL or *P*. *chabaudi* AS infection of IL-21R-/- mice. (B) Survival curve during *P*. *yoelii* XNL or *P*. *chabaudi* AS infection of C57BL/6, μMT and IL-21R-/- mice.
(TIF)

S6 Fig Increase in level of PD-L1 expression on GC B cells during lethal MHV68 and *P*. *yoelii* XNL co-infection. The timeline and experimental set up was identical to that shown in Fig 5A. (A) Absolute number of PD-L1 (B220+ GL7+ CD95+ CD274+) expressing splenic GC B cells at day 16 post-infection with *P*. *yoelii* XNL. (B) Mean Fluorescence Intensity (MFI) of the PD-L1 (CD274) marker on GC B cells at day 16 post-infection with *P*. *yoelii* XNL.
(TIF)

S7 Fig M1 does not alter kinetics of MHV68 specific IgG response during infection. C57BL/6 mice infected with 1 x 10^5^ PFU via the IN route with either the M1 null mutant (M1.Stop virus) or the marker rescue (MR) virus. Blood was collected at multiple times post viral infection. Plotted are MHV68 specific IgG responses as a function of days post viral infection. Serum from naïve mice was used as a negative control.
(TIF)
