# Supplementary figures and images for: Gammaherpesvirus Co-infection with Malaria Suppresses Anti-parasitic Humoral Immunity
Source: PLoS Pathog. 2015 May 21;11(5):e1004858. doi: 10.1371/journal.ppat.1004858 (PMC4440701; doi:10.1371/journal.ppat.1004858)

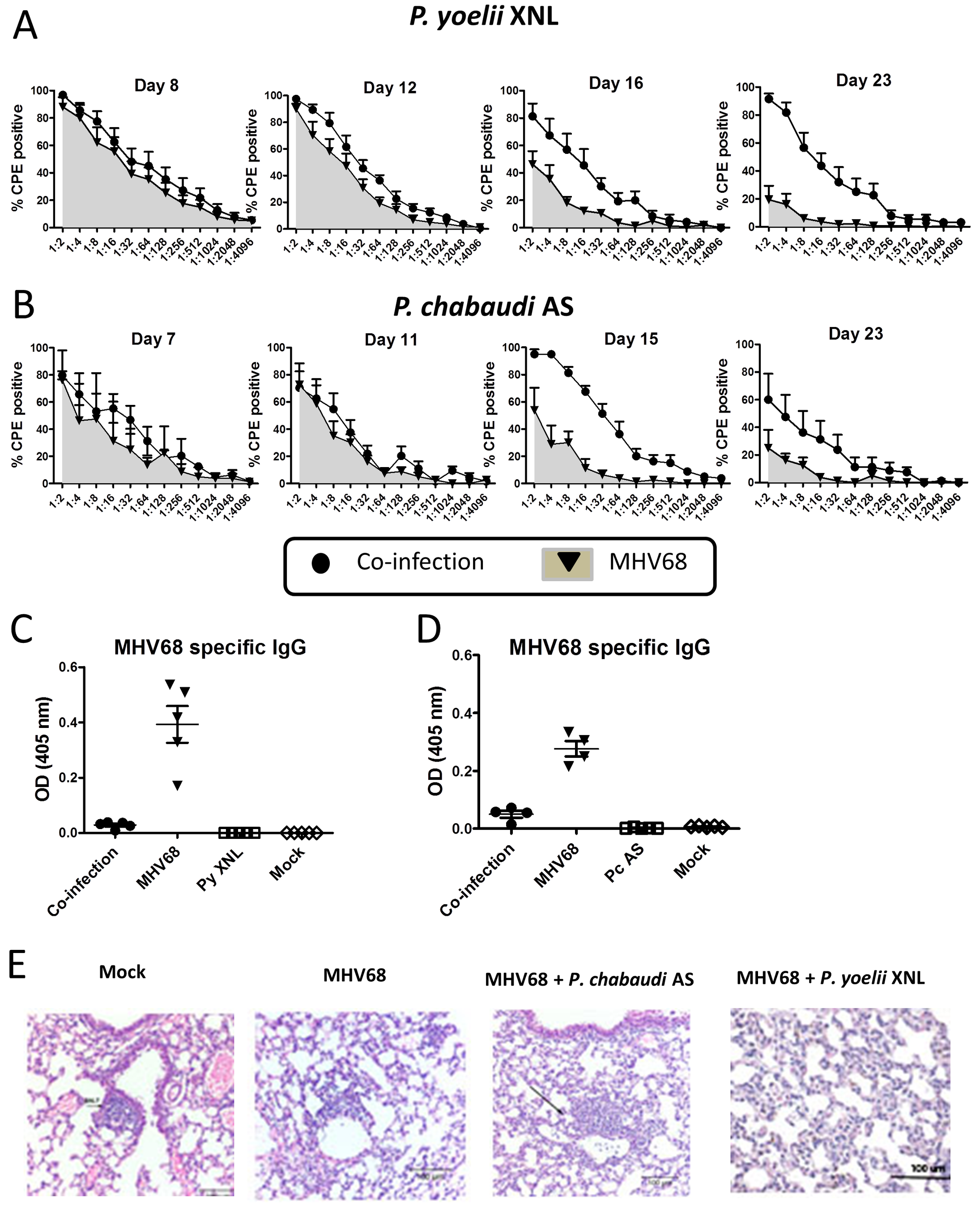

Supplement: S1 Fig — The timeline and experimental set up was identical to that shown in Fig 1A. Limiting dilution analysis of viral lung titers in the (A) P. yoelii XNL or (B) P. chabaudi AS co-infection models at multiple times post co-infection. MHV68 specific IgG titers in the serum at day 23 post Plasmodium infection in (C) P. yoelii XNL or (D) P. chabaudi AS co-infected mice. (E) Hematoxylin and eosin stain of lung tissue sections from animals sacrificed at day 23 post co-infected with MHV68 and either P. yoelii XNL or P. chabaudi AS (mock and MHV68 infected lung sections are also shown). Scale bar 100 μm. (TIF) [file ppat.1004858.s001.TIF]

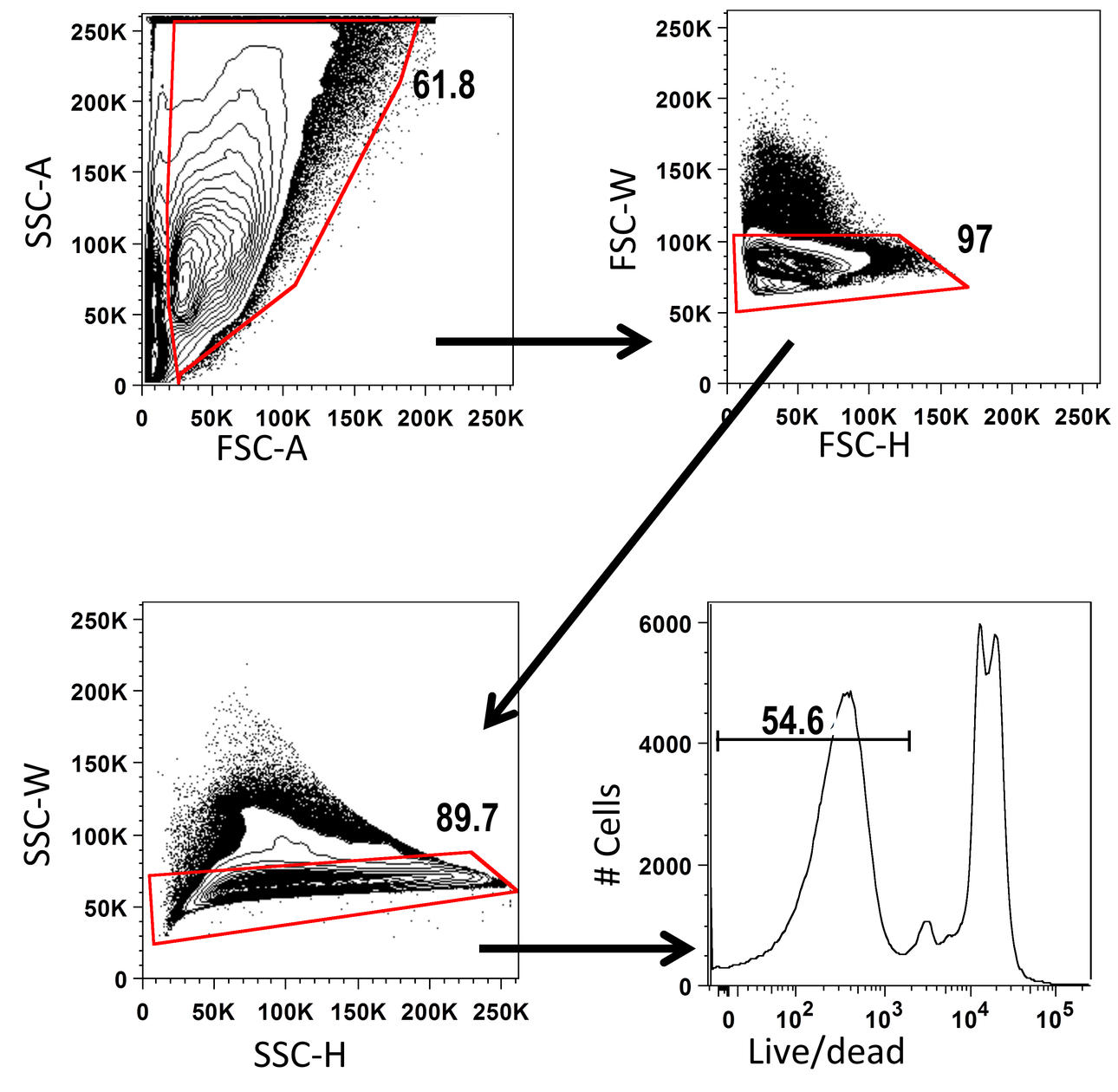

Supplement: S2 Fig — Identification of live cells was done using a fixable viability dye (Life Technologies). SSC-A, side scatter area; FSC-A, forward scatter area; FSC-W, forward scatter width; FSC-H, forward scatter height; SSC-W, side scatter width; SSC-H, side scatter height. (TIF) [file ppat.1004858.s002.TIF]

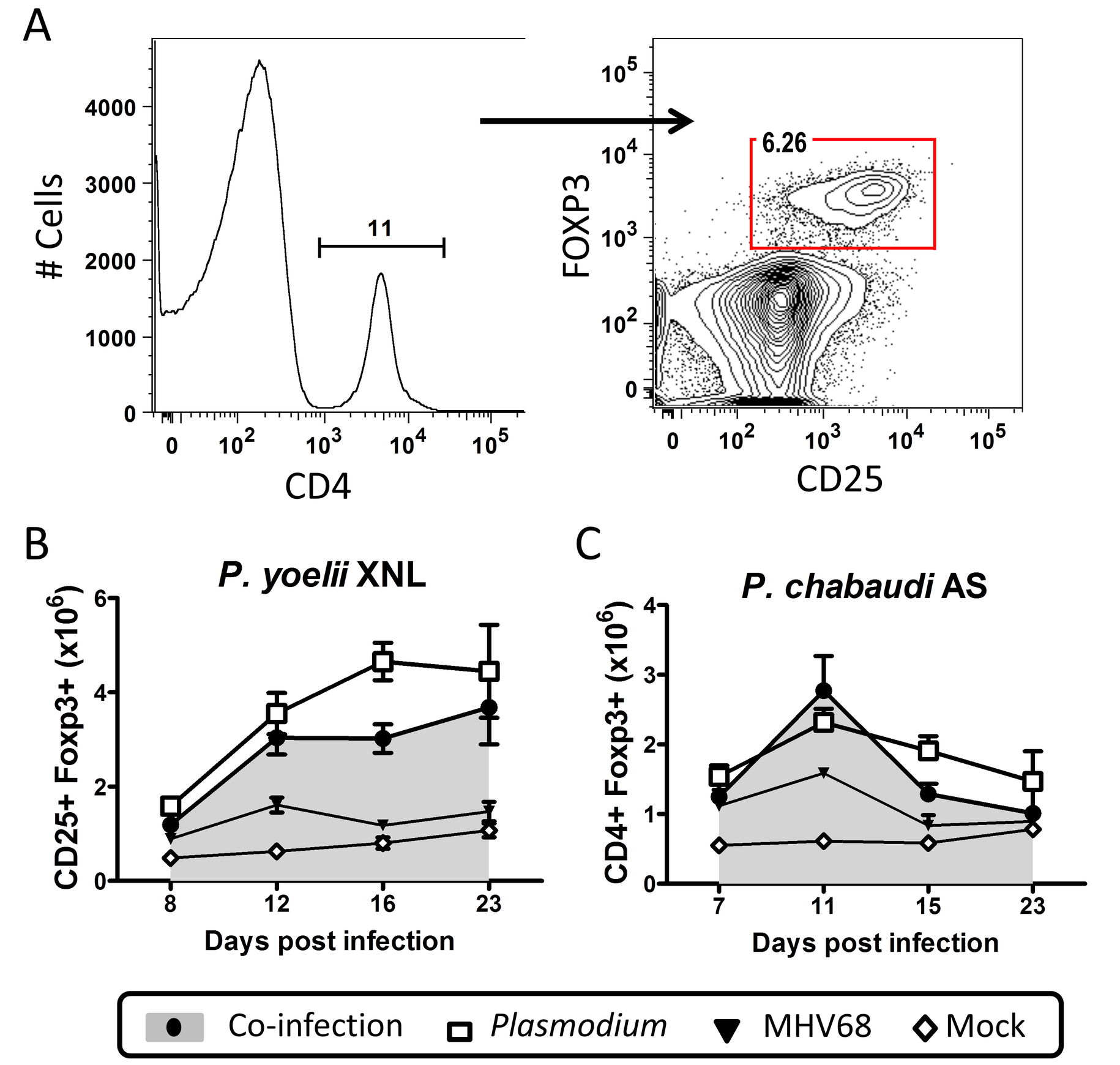

Supplement: S3 Fig — The timeline and experimental set up was identical to that shown in Fig 1A. (A) Representative flow panels show gating strategies. Cells were gated on the live population, and CD4+ cells were analyzed for cytokine production. Absolute number of CD4+ T cells producing IL-10, IFN-γ, or both in the (B) P. yoelii XNL and (C) P.chabaudi AS co-infection models. (TIF) [file ppat.1004858.s003.TIF]

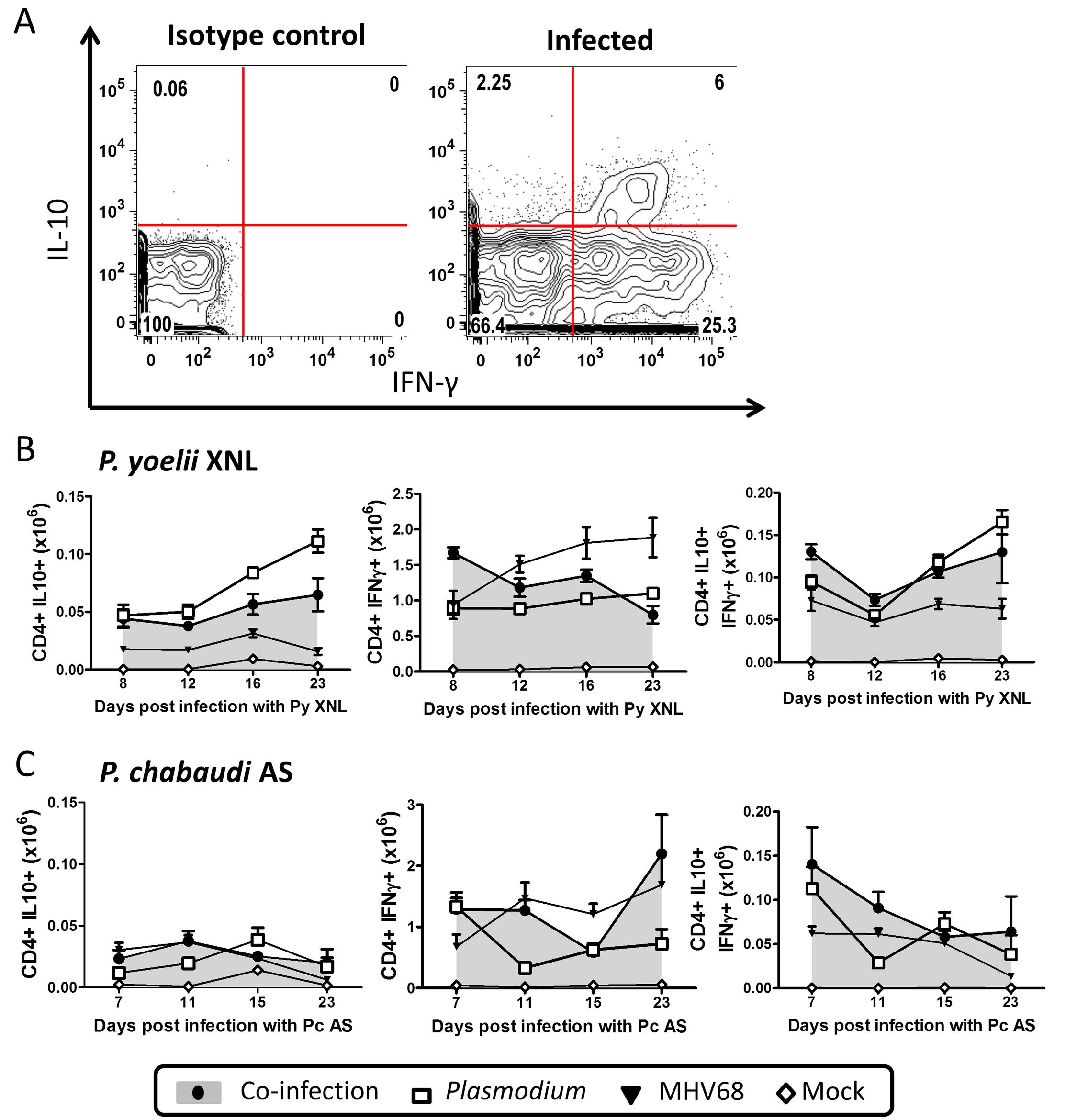

Supplement: S4 Fig — The timeline and experimental set up was identical to that shown in Fig 1A. (A) Representative flow plots showing gating strategy for Tregs (CD4+ CD25+ FoxP3+). Absolute numbers of Tregs in the spleen at indicated time points for (B) P. yoelii and (C) P. chabaudi co-infection models. (TIF) [file ppat.1004858.s004.TIF]

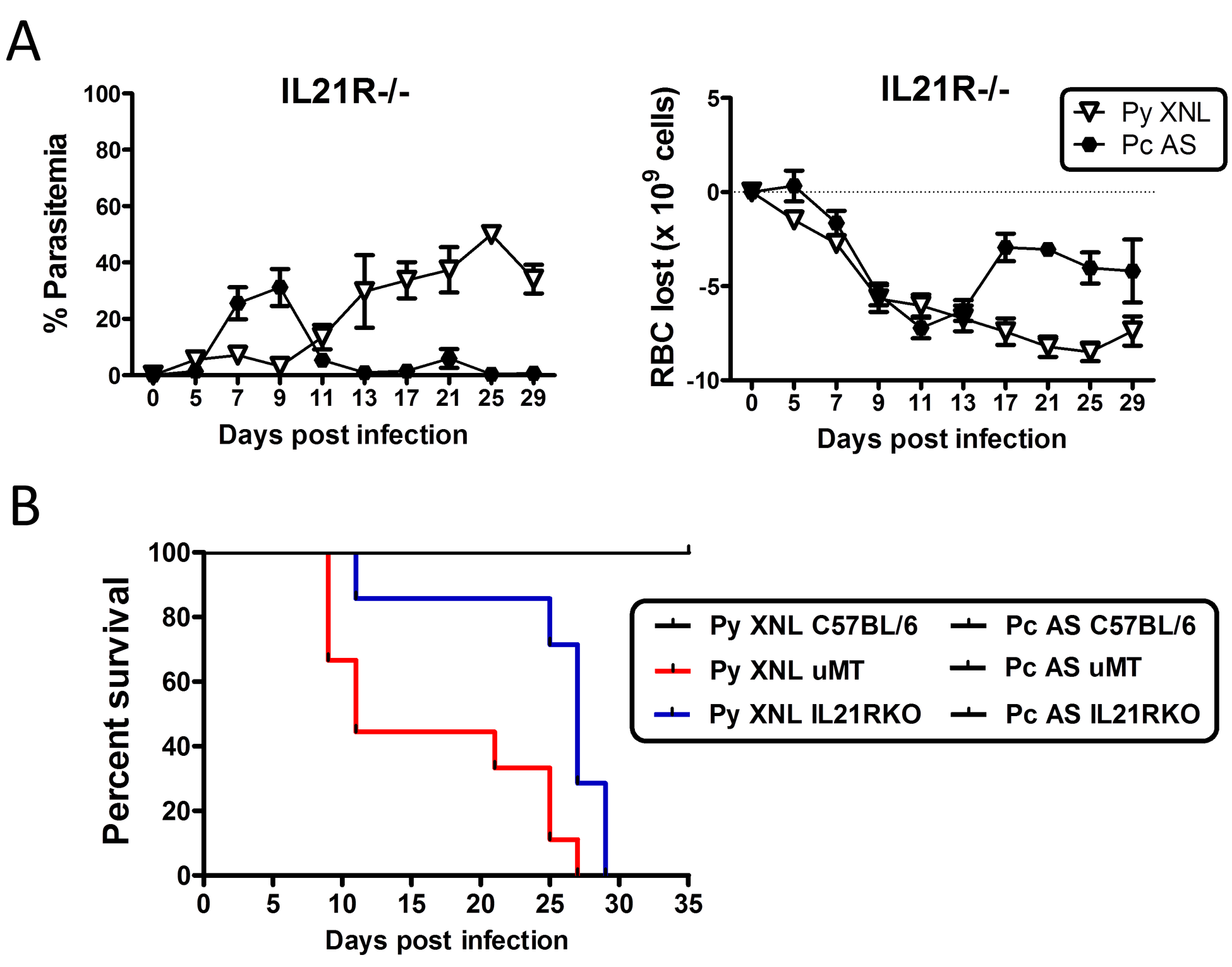

Supplement: S5 Fig — (A) % parasitemia and anemia during P. yoelii XNL or P. chabaudi AS infection of IL-21R-/- mice. (B) Survival curve during P. yoelii XNL or P. chabaudi AS infection of C57BL/6, μMT and IL-21R-/- mice. (TIF) [file ppat.1004858.s005.TIF]

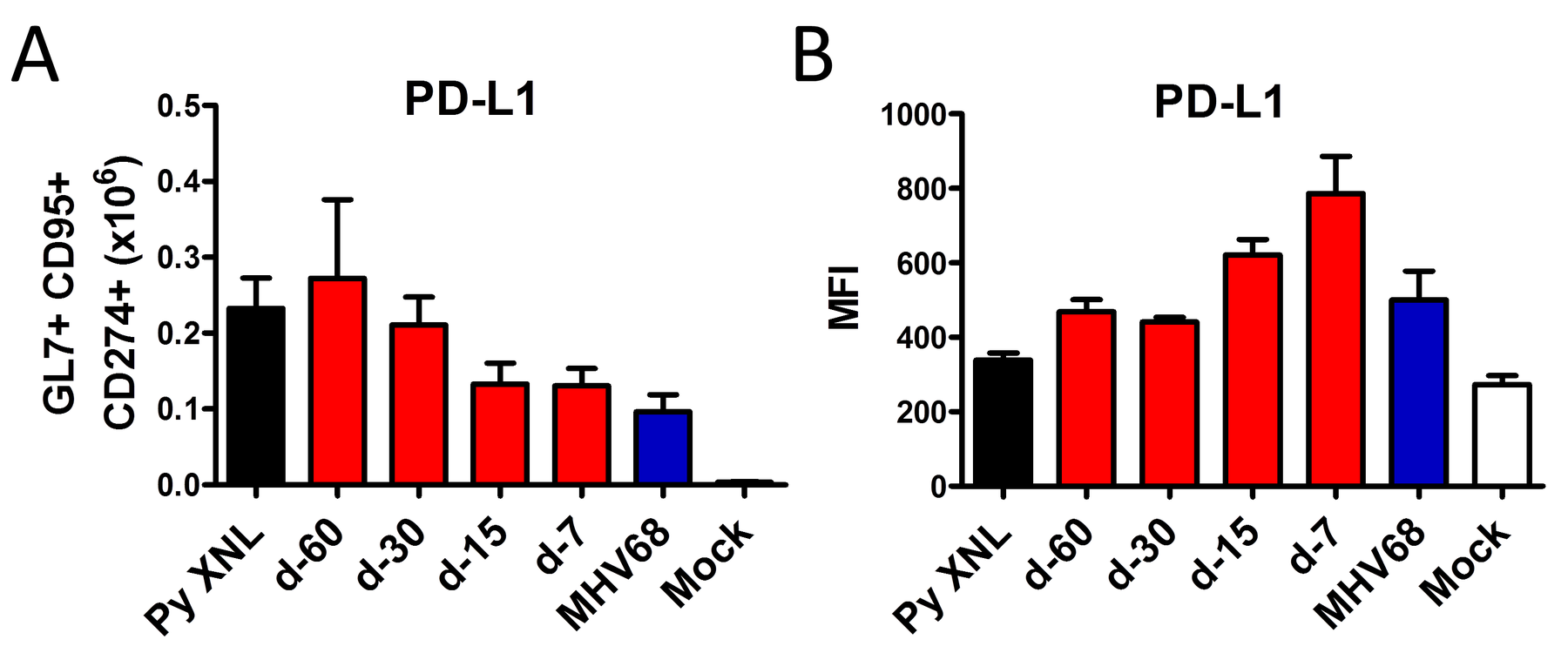

Supplement: S6 Fig — The timeline and experimental set up was identical to that shown in Fig 5A. (A) Absolute number of PD-L1 (B220+ GL7+ CD95+ CD274+) expressing splenic GC B cells at day 16 post-infection with P. yoelii XNL. (B) Mean Fluorescence Intensity (MFI) of the PD-L1 (CD274) marker on GC B cells at day 16 post-infection with P. yoelii XNL. (TIF) [file ppat.1004858.s006.TIF]

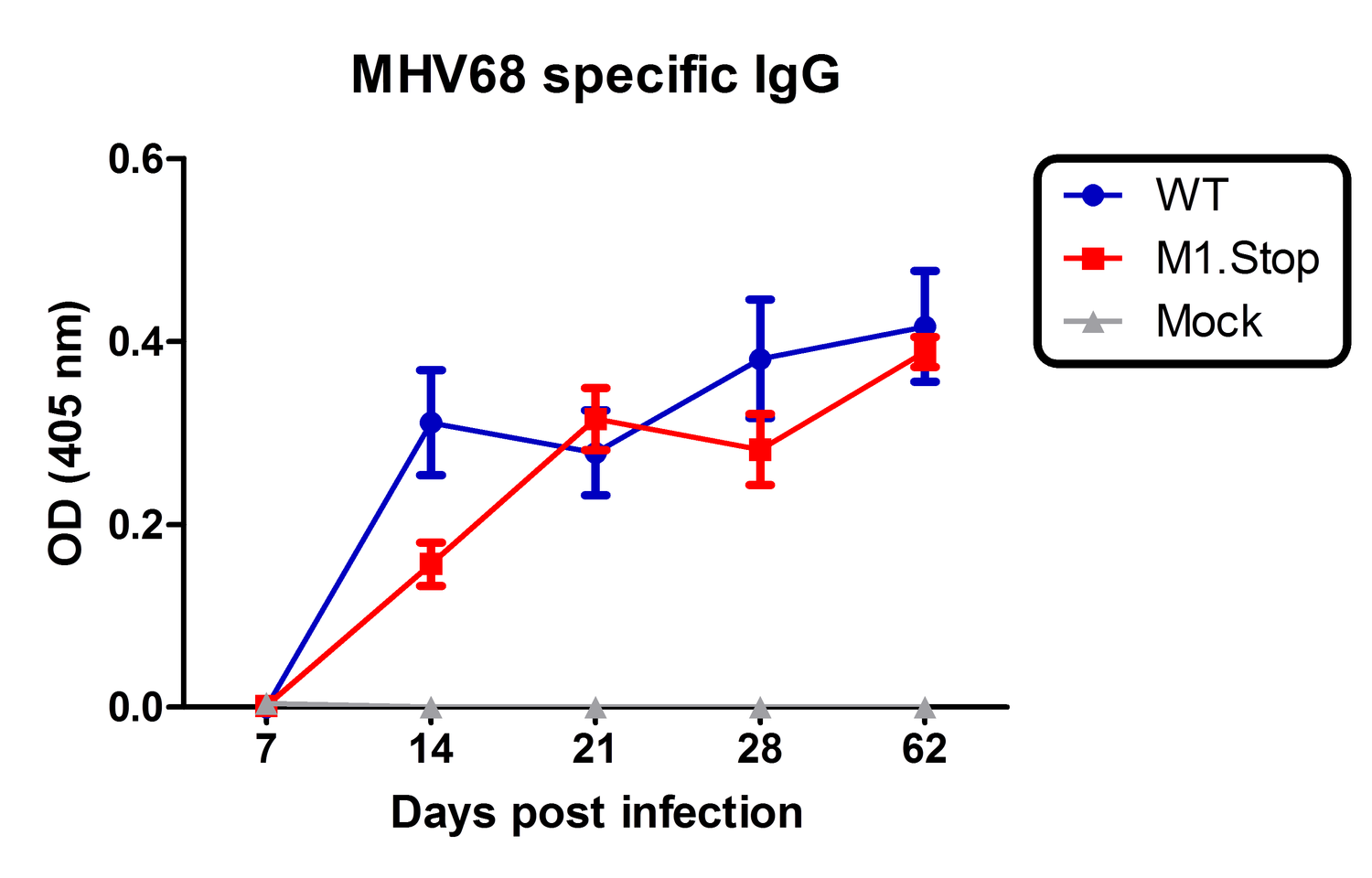

Supplement: S7 Fig — C57BL/6 mice infected with 1 x 10^5^ PFU via the IN route with either the M1 null mutant (M1.Stop virus) or the marker rescue (MR) virus. Blood was collected at multiple times post viral infection. Plotted are MHV68 specific IgG responses as a function of days post viral infection. Serum from naïve mice was used as a negative control. (TIF) [file ppat.1004858.s007.TIF]
